# Childhood as a solution to explore–exploit tensions

**DOI:** 10.1098/rstb.2019.0502

**Published:** 2020-06-01

**Authors:** Alison Gopnik

**Affiliations:** Department of Psychology, University of California, 2121 Berkeley Way, Room 3302, Berkeley, CA 94720-1650, USA

**Keywords:** development, life history, explore–exploit

## Abstract

I argue that the evolution of our life history, with its distinctively long, protected human childhood, allows an early period of broad hypothesis search and exploration, before the demands of goal-directed exploitation set in. This cognitive profile is also found in other animals and is associated with early behaviours such as neophilia and play. I relate this developmental pattern to computational ideas about explore–exploit trade-offs, search and sampling, and to neuroscience findings. I also present several lines of empirical evidence suggesting that young human learners are highly exploratory, both in terms of their search for external information and their search through hypothesis spaces. In fact, they are sometimes more exploratory than older learners and adults.

This article is part of the theme issue ‘Life history and learning: how childhood, caregiving and old age shape cognition and culture in humans and other animals’.

## 1. Introduction

One of the most distinctive aspects of human life history is our exceptionally extended childhood. Chimpanzees produce as much food as they consume by the time they are around 7 years old; even in forager cultures human children are not self-sufficient until they are at least 15 [1]. Moreover, there is evidence from the fossil record that childhood was extended during the relatively brief period in which modern humans evolved. *Homo sapiens* appear to have had a longer period of immaturity than other hominins, even including Neanderthals [2,3].

Human childhood is expensive. Adults must feed and protect the young into adolescence. Early brain development is also very energetically costly, with more than 60% of 4-year-olds' calories going to the brain at rest, compared with around 20% for adults [4]. Humans also have shorter inter-birth intervals than their closest primate relatives [5], so they stack up even more of those costly babies.

Humans developed exceptionally extended and varied sources of caregiving investment to deal with this cost, including pair-bonded fathers [6], related and unrelated alloparents [7], and post-menopausal grandmothers [8]. This is in contrast with our closest primate relatives and with most mammals. All of these types of investment are found in other species, but they are relatively rare, and they are not found in the other great apes. No other species appears to have all these types of investment. The role of grandmothers points to an additional life-history feature of humans, our longevity. We live some 20 years longer than chimpanzees.

These changes in life history evolved in concert with well-known changes in human cognitive capacities. No single psychological trait is found in humans but not in any other species, with the possible exception of language. However, the combination of brain and cognitive changes in humans is distinctive, including greatly increased brain size and neuron numbers, and an expansion of the cortex [9]. These changes also include the emergence of impressive capacities for both physical and social cognition. These include new kinds of physical tool use and physical cognition, particularly causal cognition (e.g. [10–12]), sophisticated capacities for ‘theory of mind’ and social interaction and cooperation [13–15] and impressive capacities for cultural learning and transmission [16,17].

As with caregiving investment, elements of these capacities can be found in other species. Corvids have impressive capacities for ‘folk physics’ (e.g. [18,19]). Social carnivores as well as some other primates have striking capacities for social cognition and cooperation, and recent work suggests that elements of ‘theory of mind’ can be found in great apes (e.g. [20–22]). Cetaceans such as whales and dolphins, as well as birds, show capacities for cultural learning [3,23]. But the full suite of these cognitive capacities and their extent in humans is distinctive.

The rapidity and breadth of the changes in cognition and life history that led to modern humans strongly suggests that many coevolutionary processes emerged in concert rather than that there was a single decisive adaptation. In this paper, I suggest that distinctive cognitive features of childhood, particularly the capacity and motivation for internal and external exploration in a protected period, were one important factor contributing to this coevolutionary cascade.

The fact that both the extended human childhood and the cognitive changes emerged so quickly and in concert, and particularly that the life-history changes are so costly, suggests that there is some adaptive relationship between them. This has, in fact, been suggested in a general way previously [25,26]. But what might this relationship be like in more detail? It is certainly possible that the relationship is epiphenomenal rather than adaptive. That is, a long childhood, or even simply a longer overall lifespan, might be selected for some reason and a larger brain and cortical size might simply be a result of the longer developmental period (e.g. [27]). Or, vice-versa, a larger brain might simply require a longer waiting period of development [28].

Alternatively, there might be some quite specific links between particular adult cognitive capacities and the extended human childhood. For example, Hrdy and Burkart [29], Hawkes [30] and Tomasello [31], in this volume, suggest that social cognition, in particular, was required both to allow infants and children to navigate the demands of engaging a range of carers as well as to allow multiple carers to cooperate. Alternatively, Kaplan *et al*. [1], Boyd & Richerson [16], Henrich [17] and Sterelny [32] suggest specific links between an extended childhood and time for the transmission of cultural skills, both physical and social, that are necessary for adult hunting and foraging.

However, all of these approaches assume a fundamental continuity between the minds of children and adults. I will argue instead that childhood itself, both in humans and other species, involves a distinctive set of cognitive characteristics that actually trade off with adult characteristics. This trade-off is described in the computational literature in terms of an intrinsic tension between exploration and exploitation. The ‘explore’ features of childhood minds include a general capacity for learning and plasticity which allows sensitivity to a wide range of environmental and behavioural possibilities. Moreover, this developmental programme not only involves cognitive, computational and neural differences between children and adults but also differences in motivation, emotion and action. Juveniles of many species not only show a capacity for learning about new environments, they are actively motivated to do so. This is manifested in the distinctive suite of emotions and activities that are associated with childhood, especially the neophilia and curiosity that lead to active exploration and play and are particularly characteristic of human children.

These capacities are in tension with more adult capacities for skilled action in a particular environment, including cognitive features like attentional focus, inhibition, and executive function and behaviours like long-term, goal-directed, planned action. A long childhood allows for a kind of developmental division of labour, with an early protected period devoted to learning and exploration and a later adult period devoted to directed exploitation based on what has been learned earlier.

Special capacities for learning and exploration, exercised in a protected period of immaturity, would help to enable the whole range of striking human cognitive changes. Many of the specific computations that are involved in particular cognitive capacities, such as intuitive physics or psychology, or social understanding and cooperation, could, in principle, be either built in or restricted to particular domain-specific kinds of learning (this may indeed be the case in other species). A long and wide-ranging period of learning and exploration, in contrast, would help to enable the whole range of human cognitive skills, from physics to psychology, from communication to culture.

The idea of childhood as an evolutionary solution to explore–exploit trade-offs also applies widely beyond humans, unlike some of the more specific proposals concerning cooperation and culture. It may seem evolutionarily paradoxical to develop a life history that includes expensive and vulnerable young for a long period. However, across many different species, including birds, both placental and marsupial mammals, and even insects, there is a very general (though not perfect) correlation between the length of immaturity and degree of parental investment and relative brain size, intelligence and a reliance on learning [9,33–36]. Humans are, of course, at the far end of the distribution on all these measures. Explore–exploit trade-offs may help to explain this strikingly general and widespread relationship between an extended childhood and learning.

## 2. The explore–exploit dilemma

What is the explore–exploit dilemma? Here is a classic example. You can choose whether to go to your old reliable restaurant or try a new place that might be better or might be worse. The first option will give you a good meal but no new information. The second option risks a less good meal but will give you more information—and might lead to something even better than your known choice. Which should you do? It turns out to be remarkably difficult to design a systematic strategy to solve this problem.

The most well-known versions of the problem arise in the context of reinforcement learning algorithms. These algorithms have played a major role in the recent success of machine learning, have important connections to neuroscience and have been applied extensively to foraging behaviour in non-human animals [37]. In reinforcement learning, an agent acts on the world and observes the positive or negative outcomes of those actions. The agent must choose whether to act to explore unknown properties of the environment (like trying the new restaurant) or to act to exploit known rewards (like sticking with the old favourite). For example, in a classic ‘bandit’ problem, the learner has to decide between pulling two levers. She knows that one lever will produce a reward half the time (the exploit choice), but she can also pull a new lever with an unknown pay-off (the explore choice). When should she go with the modest but sure chance of a win, and when should she risk the mystery lever that might lead to a bigger reward overall?

Solving this explore–exploit trade-off is computationally intractable in realistically complex environments, so agents must use heuristics to find an efficient solution (see [38], for a recent review). With *random* exploration heuristics, the agent might just randomly try new actions. In *directed* exploration algorithms, the agent specifically crafts actions that will be most likely to be informative. Adult humans apply a mix of random and directed exploration heuristics to solve reinforcement learning problems [39].

The classic reinforcement learning problem involves a search for new data, a kind of external exploration. But other areas of computer science, particularly optimization theory, point to a similar set of trade-offs when it comes to finding new hypotheses or solutions—a kind of internal exploration [40]. Consider, for example, a situation in which you are trying to find the best causal explanation for a particular pattern of data. If the space of possibilities is reasonably complex, it will be impossible for any system, human or computer, to consider and compare all the relevant causal hypotheses and search through the whole space. Computer scientists and statisticians often use ‘sampling’ techniques such as Monte Carlo methods to help solve this problem—semi-randomly generating some hypotheses rather than others [41]. We have discovered evidence that people, including young children, do something similar [42–44].

The search and sampling process, however, presents learners with an explore–exploit dilemma. A learner can conduct a narrow search, only revising current hypotheses when the evidence is particularly strong and making small adjustments to current theories to accommodate new evidence. This strategy is most likely to quickly yield a ‘good enough’ solution that will support immediate effective action. But it also means that the learner may fail to imagine a better alternative that is farther from the current hypothesis, such as a hypothesis about an unusual causal relation.

Alternatively, a learner can conduct a more exploratory search, moving to new hypotheses with only a small amount of evidence, and trying out potential hypotheses that are less like the current hypotheses. This strategy is less efficient if the learner's starting hypothesis is reasonably good and may mean that the learner wastes time imagining unlikely possibilities. But it may also make the learner more likely to discover genuinely new ideas. Drawing on an analogy to statistical physics, computer scientists have described this difference in terms of a contrast between narrower ‘low temperature’ exploit searches and broader ‘high temperature’ exploratory ones [40].

Although there are many different approaches to solving the explore–exploit dilemma, they tend to share a common feature—the learner should begin by exploring and gradually converge on exploiting. There are two reasons for this. One is simply that the learner is likely to start out knowing less and gradually acquire more knowledge. As the learner knows more, it makes sense to rely more on that prior knowledge and be less motivated to acquire new knowledge. The second reason is that if there is a limited period in which to solve the task, then as that period elapses, there will be fewer opportunities to take advantage of the information that has been acquired through exploration.

Similarly, in the literature on search and sampling, ‘simulated annealing’ [40] is one of the best ways of resolving the tension between high- and low-temperature strategies. Learners, who begin with a broader higher-temperature search and gradually move to a narrower low-temperature search, are most likely to find the optimal solution, just as in metallurgy heating a metal and then cooling it leads to the most robust structure.

Beginning with a high-temperature search and moving to a low-temperature one is particularly important as a way to solve the problem of ‘local optima’. In a low-temperature search, you make small changes to your current state, see if they are improvements, move to the new state if so, and then repeat the process. This is sometimes described as ‘hill-climbing’, gradually ascending to a better state (e.g. [45]). However, if the space is complex, then the current hypothesis may be better than all the local alternatives, but much worse than an alternative that is further away and unlike the current possibilities. Hill-climbing algorithms may take you to the top of a local hill but leave you stuck on a ‘local maximum’, unable to reach an even higher state on a more distant peak.

If you start off by bouncing around the space with a high degree of randomness, you will be more likely to land on this initially unlikely option, along with many others. However, in order for the solution to be stable enough to support action, the learner must eventually settle on a particular option and narrow their search. Simulated annealing, starting off hot and cooling off later, allows the learner both to escape local optima and to settle on stable solutions. In particular, the early high-temperature search allows the learner to explore the high-level structure of the problem space. It gives the learner a sense of the general kinds of solutions that are possible, while the cooler later search allows the learner to hone-in on details.

## 3. Development and explore–exploit trade-offs

My colleagues and I have argued that this explore-first/exploit-later strategy may be embodied in a developmental division of labour [46–48]. The explore–exploit division of labour might be accomplished in other ways. In the classic reinforcement learning ‘bandit’ tasks, the ‘explore then exploit’ sequence may take place during the course of learning itself, in a single individual for a single problem. In social insects, such as honeybees, different individuals may take on the exploratory versus exploitative roles—scouts contrast with worker bees. Complex human societies may do the same with specialized exploratory roles for shamans or scientists.

But the developmental life-history strategy of an extended exploratory childhood followed by an exploitative adulthood has several advantages. The interests of the child who does the learning and the grown-up who exploits what she has learned are automatically the same, since they are the same individual, obviating the free rider problems in a social division of labour. Similarly, the caregiving investment that frees up the child for exploration is linked to genetic relatedness—although alloparents may be non-kin, usually the carers are also parents, older siblings or grandparents. Moreover, the temporal sequence of development, being a child first and an adult later, automatically results in the explore-then-exploit sequence. Most of all, protected immaturity allows children to exercise specialized capacities dedicated to learning and exploration, without having to simultaneously exploit.

It may seem tautological that childhood precedes adulthood, and more general kinds of learning would precede more specific ones. But it would be possible to have the reverse developmental strategy, in which initial knowledge was highly restricted and oriented to the most significant survival challenges the organism faces, but computationally complex, well developed and highly specified. More generally applicable broad learning mechanisms such as association might be employed later to fine tune responses to a particular environment. This is the strategy implied by ‘core knowledge’ and similar nativist theories of development [49]. Indeed, arguably this is the strategy pursued by highly precocial animals such as chickens and other galliformes. These animals have elaborate but narrow computational capacities in place at birth, and use them competently (e.g. [50]). And, not coincidentally, they mature quickly, and have very brief juvenile periods.

This precocial strategy, with much structure in place early, will be especially valuable in highly constrained and predictable environments. By contrast, an extended period of exploration is particularly valuable in situations in which environments are variable, with a mix of predictability and unpredictability. There are interesting questions and significant formal work about just which kinds of variability and predictability would make extended development adaptive (see [51,52]). In general, though, the value of exploration is directly related to the dimensionality and complexity of both the environment and the potential adaptations to that environment. The broader and more complex the possibilities are, the more exploration will be valuable.

It is plausible that increased environmental variability was associated, in particular, with human evolution. The original trigger may have been increased climactic variability [53], but human behaviours themselves lead to increased environmental variability. Nomadic behaviour means that humans characteristically face novel environments and the human capacity for culture and niche construction creates novel social and physical environments across generations.

The general explore-then-exploit pattern appears to be manifested in a wide range of species with different types of learning capacities, including simpler forms of plasticity, as well as the kinds of sophisticated learning we see in humans. For example, Snell-Rood *et al*. [35] find that in cabbage white butterflies, different developmental strategies are associated with whether the animal relies on learning or is restricted to innate preferences. Individuals who rely more on learning have fewer young and a longer developmental period. Similarly, Frankenhuis & Panchanathan [52] argue that phenotypic plasticity, such as whether and when water fleas grow a protective helmet, is associated with differing developmental trajectories, and that a period of sampling the environment, determining predator density, for example, precedes the commitment to a particular specialized adaptation. ‘Sensitive periods’ are a similar example of different cognitive adaptations in different developmental periods: the animal in the sensitive period is open to learning in a particular domain, the post-sensitive animal is not. And sensitive periods usually come early in development. In general, a pattern with greater early plasticity leading to a more efficient but inflexible state later on makes sense from the explore–exploit perspective.

But the general principle applies with special force to human evolution. Primates, in general, and humans, in particular, rely on learning particularly heavily, and also have much longer childhoods and much more caregiving investment than chickens, butterflies or fleas. Rather than having a few sensitive periods of plasticity adapted to particular domains, a human mind has to explore the very wide and unpredictable range of human possibilities, both in terms of possible actions and possible hypotheses. You could think of an extended curious childhood with particularly powerful kinds of learning as a kind of turbo-powered super sensitive period—a protected time to extract information from the environment through exploration and to imagine even far-away and unlikely hypotheses.

## 4. Developmental evidence for the explore–exploit hypothesis

The explore–exploit hypothesis helps to resolve some paradoxes in children's cognitive development. Human infants and children are remarkably effective learners. In a very short time, they learn about objects, people, animals and plants creating intuitive theories of the physics, biology and psychology of the world around them. They do this well before they go to school, with no explicit teaching [13,54–56].

In the comparative literature, where most of the work on life history has taken place, there has been a tendency to contrast innate hard-wired domain-specific reflexes on the one hand, and domain-general associationist learning mechanisms on the other. But the kinds of learning that human children engage in go well beyond simple association. Even very young human children learn by formulating and testing structured causal hypotheses about the world, updating them in the light of new evidence [56–58]. Cultural learning is particularly important for human children. Children are especially sensitive to information they obtain from others, both in their everyday observation and imitation of actions and in learning from testimony (for reviews, see [59,60]).

Moreover, children do all this learning in spite of the fact that they lack many of the kinds of intelligence that are characteristic of adults. Young children are noisy, variable, unfocused, unpredictable and impulsive. They lack ‘executive function’ abilities and capacities for focused and directed attention and are notoriously bad at long-term planning and deferred gratification [61,62]. In fact, almost by definition, young children cannot even take care of themselves (this is what immaturity means).

In addition to being effective learners in general, human children are especially good at exploratory ‘active learning’ that involves the pursuit of information about the world. A new wave of empirical research shows that toddlers and preschoolers explore the world and collect new information in spontaneous, systematic and rational ways ([63–68]; see [69] for a review). Even infants systematically explore surprising events [70]. Moreover, children learn from their exploration. Bonawitz *et al*. [67] found that children revised their beliefs based on evidence they generated during exploratory play and Sim & Xu [71] found that even toddlers used the evidence they generated in exploration to formulate new abstract generalizations.

Play is intrinsically on the explore side of the explore–exploit trade-off. By definition, it involves activities that are not designed to accomplish particular goals [72]. The kind of spontaneous active learning described in the studies above is itself a form of play, but other forms of play also have wider exploratory functions. We have argued and presented data that pretend play, in particular, has a close empirical and theoretical connection to counterfactual thinking which is related to the kinds of ‘internal exploration’ that are involved in sampling and hypothesis search [11]. Schulz [69] has suggested that play might also involve a kind of ‘competence exploration’ helping children to learn what kinds of plans might solve a given class of problems.

Burghardt [72], who has extensively studied play behaviour in a wide range of animals, has argued that play can be defined as an activity that requires surplus resources but allows learning and adaptation. This fits well with the computational exploration/exploitation ideas. Childhood is a period in which extra resources are provided to an individual, and the constraints of effective and exploitative action are lifted, in order to allow learning and adaptation to the particular environment. (As Burghardt points out, sleep is another interesting example, as are the social division of labour examples described above.) Across a strikingly wide range of species, play is especially characteristic of young animals.

## 5. Evidence for an explore–exploit developmental shift

So, human children are highly effective, motivated, active and playful learners, in spite of their weaknesses in executive function. Is there evidence that they are actually more exploratory learners than adults and that these early learning abilities might actually trade off against later exploit abilities?

Comparative evidence suggests that learning, exploration and play are particularly characteristic of juveniles. Younger mice learn to reverse a learned rule more easily than post-pubertal mice [73]. Older monkeys show neural plasticity when they learn an auditory or tactile pattern, but only when the pattern is relevant to their goals—juveniles extract the patterns and demonstrate plasticity independently of goals [74]. Young capuchin monkeys are more prone to create novel behaviours relative to adults [75]. Juvenile rodents are more likely to explore aversive but informative options than adults [76]—in a classic avoidance learning task, young rats actually approach the cue that leads to a shock, preferring it to an uninformative cue, just the opposite of the adult pattern. They are especially likely to do this when the mother is present, a finding recently replicated in human preschoolers [77]. This finding also emphasizes the importance of the other half of the human life-history strategy—the expanded carer investment that allows exploration to take place.

Beyond humans, primates and rodents, there are good theoretical reasons to predict that older individuals will be less exploratory and/or neophilic than younger individuals [78], and the empirical literature, for the most part, seems to agree with this prediction (parrots and corvids: [79], geladas: [80], chimango caracara: [81–83]: great tits: [84,85], hyenas: [86]). In wild spotted hyenas, juveniles were less neophobic, more persistent and exhibited a greater diversity of initial exploratory behaviours, relative to adults, when they were presented with a puzzle box [86]. Aplin [87] found that juvenile great tits were more likely to reverse a learned rule than older individuals. Holzhaider *et al*. [88] found that New Caledonian crows used their exceptionally long fledgling period to explore the possibilities of the objects they would eventually use as tools.

Neuroscience evidence also supports the idea of an explore–exploit shift. Neuroscientists have investigated the origins of both the increased executive control and decreased plasticity that come with age. One set of developments involves synaptic changes. In the early period of development, many more new synaptic connections are made than in adulthood. With age, some of these neural connections are strengthened but others are pruned, transforming a more flexible, sensitive and plastic brain into a more effective and controlled one [89,90].

Increasing executive control in humans is also related to the development of prefrontal areas of the brain and their increasing control over other brain areas. However, neuroscientists have also argued that strong frontal control has costs for exploration and learning [91]. Interference with prefrontal control areas leads to a wider range of responses on a ‘divergent thinking’ task [92], and during learning, there is a characteristic release of frontal control [93]. All this suggests that there is a neural trade-off between increased plasticity and decreased executive function and cognitive control.

The clearest evidence for this hypothesis, however, would be evidence that human children actually perform better than adults on precisely the same tasks when those tasks involve broad search or exploration. This is a challenging empirical agenda. It is difficult to design controlled laboratory experiments to investigate capacities that are spontaneous and uncontrolled by their very nature. Experimental design almost always involves setting a goal for the participants and, by definition, exploit and executive function capacities will make participants better at achieving such goals. Indeed, adults almost always do better on laboratory tasks than children. However, there are interesting and informative exceptions to this pattern. I will outline three such studies from my laboratory in some detail and describe a number of others more briefly.

We have found that younger children show a pattern of broader hypothesis search than adults in their causal learning. Young children are more likely to infer an unusual causal hypothesis than older children and adults [46,47]. For example, the ‘blicket detector’ is a box that lights up and plays music when you put some blocks on it and not others (it is actually controlled remotely by the experimenter). The participant sees a particular pattern of activations and then must infer the causal structure of the machine, determine which blocks are ‘blickets’, and act to activate the machine themselves. Even very young children are surprisingly good at using statistical patterns to determine which particular blocks will activate the machine (see [56] for a review).

We wanted to see whether children could also infer more abstract and general causal features of the machine. Could they figure out whether the machine operated on the principle that individual blocks made it go or on the principle that combinations of blocks were required? Both children and adults initially assumed that the ‘combination’ principle was less likely than the ‘individual’ principle. Then we showed participants that individual blocks made the machine go, or that only a combination of blocks made it go. Finally, we showed them an ambiguous pattern with new blocks that was compatible with either principle, and asked them to activate the machine. Children and adults were equally good at inferring the more likely ‘individual’ hypothesis, they put the right individual blocks on the machine when that fit the data. But, to our surprise, children were substantially better at inferring the unlikely ‘combination’ hypothesis than adults—they put the right combinations of blocks on the machine when that fit the data, and the adults did not. We then replicated this pattern with low-income children and adults in Peru and low-income children in Headstart programmes in the USA [94].

Similarly, in a social causal task, younger learners were better at accurately using evidence to infer an unlikely causal hypothesis [95]. Adults, particularly in Western cultures, assume that people act the way they do because of their individual personality traits, rather than because they are faced with a particular situation, regardless of the evidence. This is ‘the fundamental attribution error’ or ‘trait bias’ [96]. We showed participants different people acting in different situations. The actions were consistent with either a trait or a situation explanation. For example, Mary might approach a trampoline and a diving board, whereas Josie avoided them (supporting a trait explanation), or Mary and Josie might both approach the trampoline and avoid the diving board (supporting a situation explanation). Then we asked participants to explain the actions—did Mary play on the trampoline because she was brave or because the trampoline was safe? Although the task is quite different from the ‘blicket detector’ task, it also involves inferring a very general and abstract causal schema from the data. And we found a similar result. Preschoolers gave the explanation that was the best fit to the data. But 6-year-olds, like adults, gave trait explanations even when the data suggested a situation explanation.

In a further study [48], we extended the range of ages we studied across the whole period of childhood from preschool through school age and adolescence. Just as children may have cognitive abilities that are qualitatively different from those of adults, different developmental periods may be qualitatively different. Until about 5, children contribute very few resources but make their greatest advances in learning. Around 5–7, there is a qualitative change. Children in contemporary Western society begin school, and in other societies and historical periods, begin more active apprenticeship and involvement in adult work. Another qualitative change comes at adolescence, which is a period of renewed exploration, risk-taking and learning, particularly in the social sphere [97].

We found qualitative changes between the preschool and school-aged children, and between school age and adolescence, but continuity within the school-age period. Interestingly, the changes went in opposite directions for the physical and social causal tasks. There was a qualitative shift at adolescence; like adults, teenagers became much less likely to infer the unlikely physical hypotheses. However, adolescents were actually *more* likely to infer the unlikely social hypothesis than either school-aged children or adults and were as cognitively flexible as the preschoolers.

There is also increasing recent evidence that younger learners prefer to explore rather than exploit and that this enables wider learning. In a very recent study [98], we gave participants a reinforcement learning task in which they could sequentially choose whether to place blocks on a machine. We told them that some blocks would lead to rewards and others to costs but did not reveal what differentiated the two. The actual rule was two-dimensional (e.g. black striped blocks were costly but white striped blocks or black spotted blocks led to rewards). After one negative trial, adults quickly assumed the most obvious rule, that a single feature differentiated the blocks (e.g. all black blocks were costly), and so avoided all the blocks with that feature. But this meant that they never received evidence that showed that the actual rule was more complex and so failed to learn the correct rule. As in other studies, they fell into a ‘learning trap’ [99]. Preschoolers, in contrast, continued to try all the blocks on the machine, and so learned the rule correctly. There is other very recent similar evidence that children explore more than adults in reinforcement learning tasks and learn more as a result, even though they may incur costs to do so [100–102].

In this research, children appear to have the greatest advantage over adults when they must infer hypotheses that have an unusual abstract high-level structure. This makes sense from a computational perspective. High-level abstract schemas typically constrain lower-level hypotheses and shape learner's interpretation of the data [103]. As learners grow older, these schemes are increasingly well confirmed and become harder to overturn, even though learners may make revisions at the lower level.

There is other evidence for relevant differences between younger and older learners. Younger learners are more able to learn new linguistic distinctions than older learners [104,105] and they are better at imagining new uses for a tool [106]. Younger children also remember information that is outside the focus of goal-directed attention better than adults and older children [107].

Humans are a cultural species, with variable and dynamic environments. For such a species, it may be particularly important for each new generation to quickly discover novel abstract features of the environment. Cultural learning risks a particular kind of local optima, the result of the intrinsic tension between imitation and innovation. Adults who are faced with a changing social or physical environment may only be willing to make small local changes to the representations they have already learned and that support their actions and plans. The young children of the next generation, in contrast, may be more willing to consider a variety of high-level schemes to explain the data they see, allowing them to eventually make broader and more accurate predictions. To take two contemporary examples, my own computer schema involves a keyboard interface, with exceptions for touch and voice, and my marriage schema involves heterosexual couples with the exceptional addition of gay marriage. By contrast, my preschool grandchildren assume that computers generally work by touch and voice and that gender is irrelevant to marriage and make different predictions and produce different actions as a result. This ability to discover novel high-level features of the environment at an early age, and so to shape further learning, could provide considerable advantages later on, in the exploit phase (see [48] for further discussion).

We still have not discovered the full extent and nature of children's exploratory behaviour and there may well be contexts where adults are able to explore more widely than children. However, many adult types of exploration, such as those involved in formal and informal science and technology, actually also require exploit abilities. In particular, there is evidence that both human and primate juveniles are less able to actually produce useful new tools than adults [108,109], although younger children are better able to imagine new uses for an existing artefact than older ones [106]. This task and other classic adult ‘insight’ tasks require both explore and exploit abilities. Actually creating an effective new tool requires both the ability to imagine alternatives to the current options and the ability to select which of those options is most likely to be effective, as well as to realize that option. These are classic exploit abilities that characteristically require executive function.

‘Over-imitation’ is another example of a behaviour that appears to suggest that children explore more narrowly than adults. When children imitate complex tool-use behaviours, they often include even unnecessary details, initially suggesting that they only consider a narrow range of options for action [110]. More recent studies, however, have shown that over-imitation actually reflects more sophisticated inferences about the physical and social world. Over-imitation varies depending on how much children know about the physical causal relations in the task [111], and on whether the demonstrator's action is accidental, intentional or pedagogical [112] and it varies depending on the social context, for example, children are more likely to over-imitate when the demonstrators are present and when actions are arbitrary and might be interpreted as rituals [113,114] (see review in [60]). Over-imitation does not appear to emerge because children have an automatic impulse to narrowly reproduce the actions of others. Instead, it appears to stem from the fact that children believe that the demonstrator is a knowledgeable expert from their own group attempting to teach them about a tool or instructing them in a social ritual. Insofar as children learn from a range of demonstrators and combine that information with their own experiences and inferences, imitation may actually help them consider a broader rather than narrower range of possibilities, though further studies are necessary to determine if this is true.

## 6. Conclusion

The explore/exploit trade-off helps to make sense of the general and widespread relationship between life history and learning. Across many different kinds of organisms, the length of immaturity and the complexity and flexibility of behaviour and cognition are correlated. The idea is particularly applicable, however, to human evolution, which involved particularly dramatic changes on both these dimensions. To return to the introduction, it is highly plausible that human evolution involved coevolutionary cascades between multiple changes in life history, behaviour and cognition. For example, a single change in the unfolding of the entire lifespan could have simultaneously allowed a long period of childhood exploration, larger brain size, more possibilities for cultural learning and a wider range of carers, particularly elders such as grandmothers, all interacting in a positive evolutionary feedback loop. Language, in turn, may have magnified all these changes. But the exploratory character of childhood cognition may have played an important role. Certainly, more empirical evolutionary and computational investigations of the links between childhood, learning, exploration and play would be illuminating.

## Data accessibility

This article has no additional data.
